# Rare Species-Driven Diversity–Ecosystem Multifunctionality Relationships are Promoted by Stochastic Community Assembly

**DOI:** 10.1128/mbio.00449-22

**Published:** 2022-04-14

**Authors:** Zhengqing Zhang, Yahai Lu, Gehong Wei, Shuo Jiao

**Affiliations:** a State Key Laboratory of Crop Stress Biology in Arid Areas, Shaanxi Key Laboratory of Agricultural and Environmental Microbiology, College of Life Sciences, Northwest A&F University, Yangling, Shaanxi, People’s Republic of China; b College of Urban and Environmental Sciences, Peking University, Beijing, People’s Republic of China; Corporación CorpoGen

**Keywords:** BEF relationships, rare species, functional role, community assembly, BEF

## Abstract

The relative functional importance of rare and abundant species in driving relationships between biodiversity and ecosystem functions (BEF) remains unknown. Here, we investigated the functional roles of rare and abundant species diversity (multitrophic soil organism groups) on multifunctionality derived from 16 ecosystem functions in 228 agricultural fields relating to soil and crop health. The results revealed that the diversity of rare species, rather than of abundant species, was positively related to multifunctionality. Abundant taxa tended to maintain a larger number of functions than rare taxa, while rare subcommunity contributed more phylotypes supporting to the single ecosystem functions. Community assembly processes were closely related to the ecosystem functional performance of soil biodiversity, only observed in rare subcommunity. Higher relative contributions of stochastic assembly processes promoted the positive effects of diversity of rare taxa on multifunctionality, while reducing their diversity and multifunctionality overall. Our results highlight the importance of rare species for ecosystem multifunctionality and elucidate the linkage between ecological assembly processes and BEF relationships.

## INTRODUCTION

Biodiversity influences ecosystem functioning (1). Over the past 2 decades, research on the links between biodiversity and ecosystem functions has focused mainly on aboveground organisms (2–4). Belowground organisms are extremely complex and diverse (5), and participate in fundamental ecosystem functions, such as organic carbon turnover, nutrient-use efficiency, pathogen control, and productivity. These functions are closely linked to climate regulation, global food supply, and other ecosystem services (4–8). Mounting evidence, obtained via controlled experiments (9, 10) and large-scale observational surveys (2–4), suggests that soil biodiversity could enhance ecosystem multifunctionality. However, it has been argued elsewhere that it is not the total number of species *per se*, but the functional properties of specific species, that drive ecosystem functioning (11, 12). Ecological communities commonly exhibit unbalanced distributions, containing a large number of low-abundance taxa and a small number of high-abundance taxa (13, 14). Current studies measuring multifunctionality in terrestrial ecosystems urgently need to incorporate comprehensive estimations of the relative functional importance of these different species.

The highly abundant species which account for the majority of biomass could occupy diverse niches, competitively utilize an array of resources, and effectively adapt to various environments (13, 15, 16). They are thought to be important in driving ecosystem functioning (17, 18). The low-abundance taxa are often referred to as the “rare biosphere” (17, 19). Previous studies have shown that rare species of macroorganisms, such as grasses and fish, play a crucial role in affecting several ecosystem functions (11, 20, 21). Rare biospheres comprise the overwhelming majority of species in natural communities and are more sensitive to environmental disturbances (22, 23). Recent studies have increasingly emphasized the importance of rare species (24) in some specialized functions, such as sulfate reduction (25) and phenanthrene degradation (26), as well as in mediating ecosystem stability and function (27, 28). In these cases, we hypothesized that rare and abundant taxa would have fundamentally different influences on ecosystem functioning; in particular, abundant species would exhibit broad functional performance, whereas rare species would universally display narrower functional roles.

Understanding the relationship between biodiversity and ecosystem functioning is a central issue in ecology that contributes to the better provisioning of key ecosystem services for humans (29). The relationships between biodiversity and ecosystem functioning have been shown to change with abiotic conditions, land use intensification (3, 30–33), the identity and number of functions (34, 35), and the type of organism being considered (3, 4, 30). It is well recognized that the strength of a biodiversity-ecosystem function (BEF) relationship is determined by three mechanisms: functional redundancy, complementarity, and species competition (29, 36–40). For example, the loss of one or a few species in a community with high functional redundancy could have minimal consequences on ecosystem function (38). Meanwhile, the complementarity effect describes the idea that niche differentiation or facilitation between species can increase the functional performance of communities (39). Finally, species competition for resources can reduce community functioning when species that perform major functions are inhibited (36). Meanwhile, community assembly processes are unexpectedly neglected when investigating the determinants of BEF relationships. Uncovering community assembly processes is critical for understanding biodiversity generation and its contribution to ecosystem functions (41–44). For example, stochastic assembly processes have been shown to be dominant in communities with more positive species covariations, which might consequently increase the functional performance of the community (45, 46). In addition, deterministic processes may reduce the effect of biodiversity on ecosystem function via dilution, if the species selected are not the ones which affect ecosystem functioning (43). Thus, the second hypothesis was that a higher relative contribution of stochastic assembly processes would promote the positive effect of diversity on multifunctionality.

This study had two main aims. The first aim was to assess the effects of the diversity of rare and abundant belowground species on multifunctionality. Multifunctionality was assessed by considering 16 ecosystem functions related to nutrient provisioning, element cycling, pathogen control, and plant-microbe symbiosis. The second aim was to explore whether and how community assembly processes influence the strength of BEF relationships. Biodiversity information was obtained for soil archaea, bacteria, fungi, and protists from 228 agricultural fields across eastern China. A recent sibling study demonstrated the importance of soil biodiversity in supporting and maintaining ecosystem functioning in agricultural systems (47). This study placed a focus on the different functional roles of rare and abundant species diversities on multifunctionality, and on the influence of community assembly in typical human-managed agricultural ecosystems.

## RESULTS

**FIG 1:**
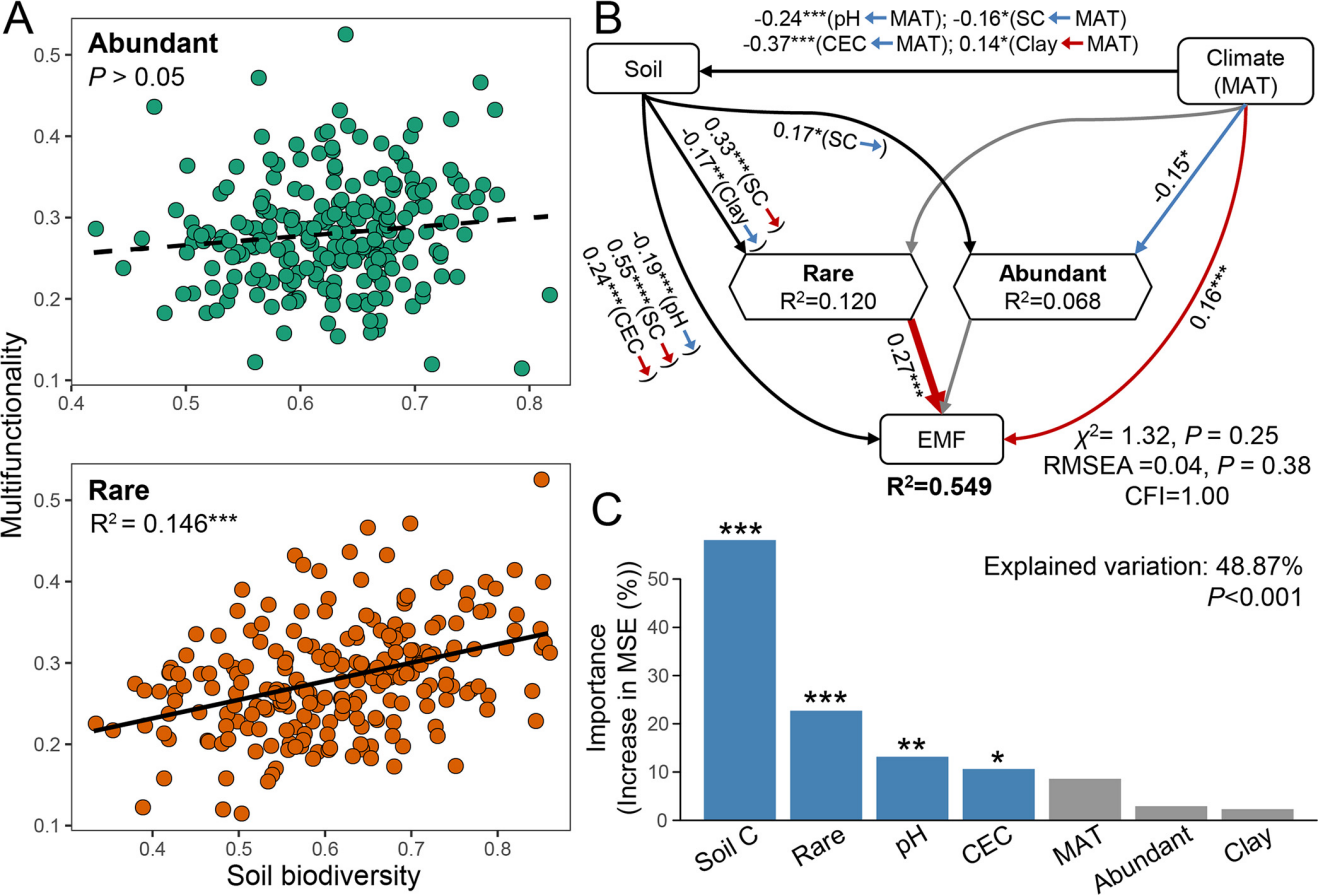
Effects of the biodiversity of rare and abundant species on multifunctionality. (A) Linear relationships between multifunctionality and the biodiversity of rare and abundant species. Statistical analysis was performed using ordinary least-squares linear regressions; *P* values are indicated by asterisks: ***, *P* < 0.001. (B) Structural equation model describing the direct relationship between the biodiversity of rare and abundant species and averaging ecosystem multifunctionality (EMF). We grouped the edaphic properties into the same box in the model for graphical simplicity, which does not represent latent variables. Numbers adjacent to arrows indicate the size of the relationship effect. R^2^ denotes the proportion of variance explained. Red arrows represent significantly positive paths, and blue arrows representsignificantly negative paths. R^2^ denotes the proportion of variance explained. Significance levels are shown as follows: *, *P* < 0.05; **, *P* < 0.01; ***, *P* < 0.001. RMSEA, root mean square error of approximation; CFI, Comparative Fit Index; MAT, mean annual temperature; CEC, cation exchange capacity. (C) Random forest mean predictor importance of influencing factors for multifunctionality. Accuracy importance measure was computed for each tree and averaged over the whole forest (5,000 trees). Percentage increases in the MSE (mean squared error) of variables were used to estimate the importance of these predictors, with higher MSE% values implying more important predictors.

We first estimated the effects of the diversities of abundant and rare soil taxa on ecosystem multifunctionality. Here, operational taxonomic units (OTUs) with relative abundances above 0.5% of the total sequences were defined as “abundant” taxa; those with relative abundances below 0.05% were defined as “rare” taxa. A significant and positive relationship between soil biodiversity and averaging multifunctionality was observed for rare taxa, but not for abundant taxa (Fig. 1A). We further compared the BEFs for rare, intermediate (OTUs with relative abundances between 0.05% and 0.5%) and abundant subcommunities, as well as for the whole community. The results showed that the diversity of rare taxa contributed the most toward explaining variation in multifunctionality (Fig. S1A), indicating that the diversity of rare taxa was truly an exceptional indicator of multifunctionality. We further explored the relationships between different ecosystems and individual soil organisms and observed that the diversity of rare taxa exhibited greater positive associations with multifunctionality than that of abundant taxa (Fig. S1B). A structural equation model (SEM) was then constructed to quantify the contributions of the diversities of abundant and rare taxa to ecosystem multifunctionality, while accounting for multiple multifunctionality drivers simultaneously. These drivers included climate (mean annual temperature [MAT]) and soil properties (soil pH, total C, and percentage of clay). The positive and direct effect of rare soil taxa diversity on multifunctionality was maintained after accounting for these other key factors (Fig. 1B). This was verified based on the strong goodness-of-fit of the SEM. The diversity of abundant taxa had no significant effect on multifunctionality. The random forest (RF) model further confirmed these observations (Fig. 1C). Moreover, these results were maintained when applying an alternative multifunctionality index weighted by four groups of ecosystem services (nutrient provisioning, element cycling, pathogen control, and plant-microbe symbiosis [abundance of soil ectomycorrhizal fungi]). The weighting was performed in a way which ensured that the functions associated with each ecosystem service contributed equally to multifunctionality (Fig. S2). These results were also maintained when applying multidimensional functionality (Fig. S3).

10.1128/mbio.00449-22.1 FIG S1 (A) Explaining variations in rare, intermediate, and abundant subcommunities, as well as in the whole community, in multifunctionality. ***, *P* < 0.001. (B) Functional effects (standardized slopes [mean ± standard error of the mean]) of the biodiversity of rare and abundant species on multifunctionality in different ecosystems (top panel) and for individual soil organisms (bottom panel). Download FIG S1, TIF file, 0.7 MB. Copyright © 2022 Zhang et al. 2022 Zhang et al. https://creativecommons.org/licenses/by/4.0/ This content is distributed under the terms of the Creative Commons Attribution 4.0 International license.

10.1128/mbio.00449-22.2 FIG S2 Effects of the biodiversity of rare and abundant species on weighted multifunctionality. (A) Linear relationships between multifunctionality and the biodiversity of rare and abundant species. Statistical analysis was performed using ordinary least squares linear regressions; *P* values are indicated by asterisks: ***, *P* < 0.001. (B) Structural equation model describing the direct relationship between the biodiversity of rare and abundant species and the average ecosystem multifunctionality (EMF). We grouped edaphic properties into the same box in the model for graphical simplicity, which does not represent latent variables. Numbers adjacent to arrows indicate the size of the relationship effect. R^2^ denotes the proportion of variance explained. Red arrows represent significantly positive paths, and blue arrows represent significantly negative paths. Significance levels were as follows: *, *P* < 0.05; **, *P* < 0.01; and ***, *P* < 0.001. RMSEA, root mean square error of approximation; CFI, Comparative Fit Index; MAT, mean annual temperature; CEC, cation exchange capacity. (C) Random forest mean predictor importance of influencing factors for multifunctionality. Accuracy importance measure was computed for each tree and averaged over the whole forest (5,000 trees). Percentage increases in the MSE (mean squared error) of variables were used to estimate the importance of these predictors, with higher MSE% values implying more important predictors. Download FIG S2, TIF file, 0.7 MB. Copyright © 2022 Zhang et al. 2022 Zhang et al. https://creativecommons.org/licenses/by/4.0/ This content is distributed under the terms of the Creative Commons Attribution 4.0 International license.

10.1128/mbio.00449-22.3 FIG S3 Effects of the biodiversity of rare and abundant species on multifunctionality, represented by the first axes from a multidimensional scaling analysis. (A) Linear relationships between multifunctionality and the biodiversity of rare and abundant species. Statistical analysis was performed using ordinary least squares linear regressions; *P* values are indicated by asterisks: ***, *P* < 0.001. (B) Structural equation model describing the direct relationship between the biodiversity of rare and abundant species and the average ecosystem multifunctionality (EMF). We grouped edaphic properties into the same box in the model for graphical simplicity, which does not represent latent variables. Numbers adjacent to arrows indicate the size of the relationship effect. R^2^ denotes the proportion of variance explained. Red arrows represent significantly positive paths, and blue arrows represent significantly negative paths. Significance levels are as follows: *, *P* < 0.05; **, *P* < 0.01; and ***, *P* < 0.001. (C) Random forest mean predictor importance of influencing factors for multifunctionality. Accuracy importance measure was computed for each tree and averaged over the whole forest (5,000 trees). Percentage increases in the MSE of variables was used to estimate the importance of these predictors, with higher MSE% values implying more important predictors. Download FIG S3, TIF file, 0.7 MB. Copyright © 2022 Zhang et al. 2022 Zhang et al. https://creativecommons.org/licenses/by/4.0/ This content is distributed under the terms of the Creative Commons Attribution 4.0 International license.

**FIG 2:**
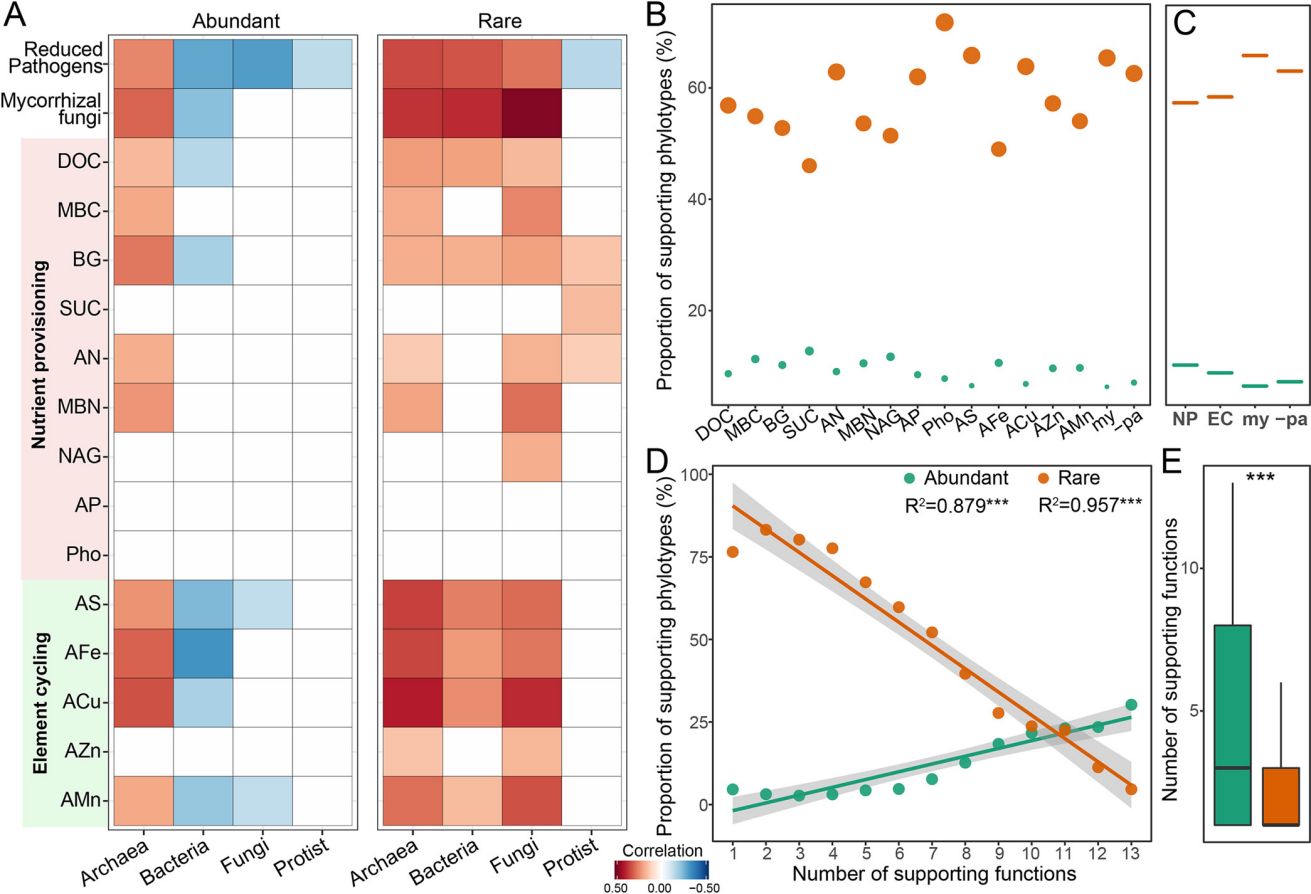
Functional role of the diversity of rare and abundant species on multifunctionality. (A) Significant correlations (Spearman; *P* < 0.05) between the diversities of single organism groups and single ecosystem functions. (B) Proportions of phylotypes supporting the single ecosystem functions. We defined phylotypes which significantly and positively correlated with ecosystem functions as “supporting phylotypes.” (C) Proportions of phylotypes supporting for different groups of ecosystem functions, including functions relating to nutrient provisioning (“NP”), element cycling (“EC”), pathogen control (“−Pa”), and plant-microbe symbiosis (“my”). (D) Linear relationships between the proportions of supporting phylotypes and the number of their supporting functions. (E) Difference in the average number of supporting functions between abundant and rare phylotypes (***, *P* < 0.001; Wilcoxon rank-sum test).

We then explored the effects of rare and abundant taxa on single ecosystem functions. The diversity of most single groups of rare taxa exhibited significantly positive relationships with most single ecosystem functions, which was not the case for the abundant taxa (Fig. 2A). To explore the effects of rare and abundant taxa on ecosystem functions, we estimated the positive effects of individual phylotypes on single ecosystem functions. The results showed that most of the supporting phylotypes for the single ecosystem functions belonged to rare taxa, rather than abundant ones (Fig. 2B). In addition, the proportion of supporting rare phylotypes was higher for the functions related to pathogen control and plant-microbe symbiosis. In contrast, the abundant taxa mainly supported functions relating to nutrient provisioning and element cycling (Fig. 2C). We further examined the relationships between the proportion of supporting phylotypes and the number of their supporting functions. Significant positive and negative relationships were observed for abundant and rare taxa, respectively (Fig. 2D). In addition, the supporting abundant phylotypes supported greater numbers of functions than the supporting rare phylotypes (Fig. 2E). This indicated that the individual supporting abundant phylotypes tended to maintain more functions than the rare taxa, while the rare subcommunity contributed more phylotypes which supported single ecosystem functions. Given that rare taxa contributed most of the total biodiversity (Fig. S4A), it was observed that the proportion of supporting phylotypes within the abundant subcommunity was still higher than that within the rare subcommunity (Fig. S4B).

10.1128/mbio.00449-22.4 FIG S4 (A) Difference in the proportions of abundant and rare taxa in each sample. (B) Proportions of phylotypes which supported single ecosystem functions within the abundant and rare subcommunities. Download FIG S4, TIF file, 0.6 MB. Copyright © 2022 Zhang et al. 2022 Zhang et al. https://creativecommons.org/licenses/by/4.0/ This content is distributed under the terms of the Creative Commons Attribution 4.0 International license.

**FIG 3:**
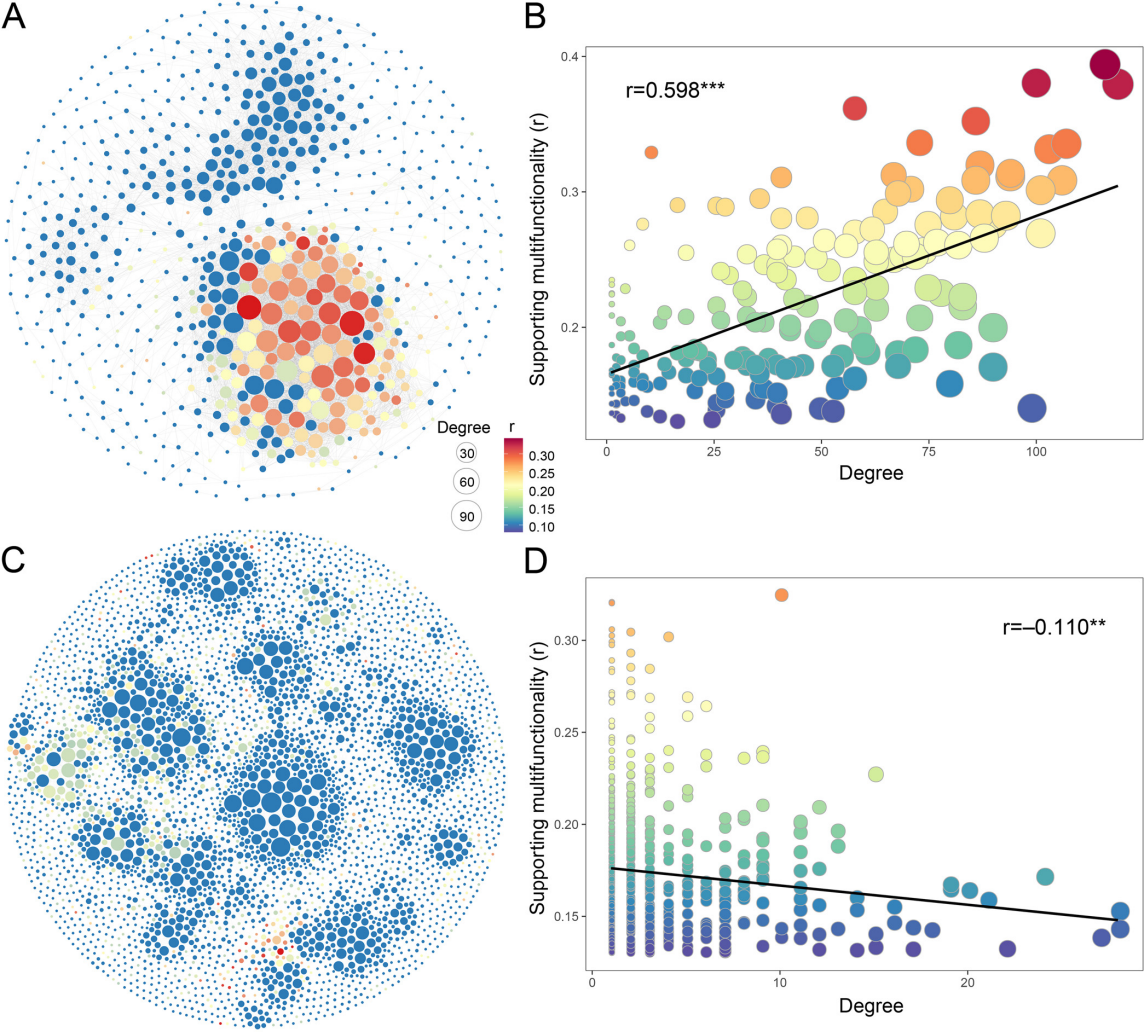
Links between the associations among soil phylotypes and their support for multifunctionality. (A and C) Correlation networks for abundant (A) and rare (C) taxa. Networks are colored based on the Pearson’s correlation coefficients between each individual phylotype and multifunctionality, which were used to reflect their support for multifunctionality. (B and D) Linear relationships between the nodes’ degree and their support for multifunctionality of abundant (B) and rare (D) taxa. **, *P* < 0.01; ***, *P* < 0.001.

Correlation networks were established for abundant and rare soil taxa to explore the strong associations among soil phylotypes of different organism groups and their contributions to multifunctionality. The abundant taxa that were more strongly correlated with multifunctionality, with higher degrees in the correlation network (Fig. 3A). The opposite trend was observed in the rare-taxa networks (Fig. 3C). These were verified by significant linear regression relationships (Fig. 3B and D). These results imply that there were distinct interaction strategies between abundant and rare taxa in supporting multifunctionality. These observations were maintained when alternative multifunctionality indices were applied (Fig. S5).

10.1128/mbio.00449-22.5 FIG S5 Linear relationships between the nodes’ degree and their supporting for weighted (A and B) and multidimensional (C and D) multifunctionality of abundant (A and C) and rare (B and D) taxa. **, *P* < 0.01; ***, *P* < 0.001. Download FIG S5, TIF file, 1.9 MB. Copyright © 2022 Zhang et al. 2022 Zhang et al. https://creativecommons.org/licenses/by/4.0/ This content is distributed under the terms of the Creative Commons Attribution 4.0 International license.

**FIG 4:**
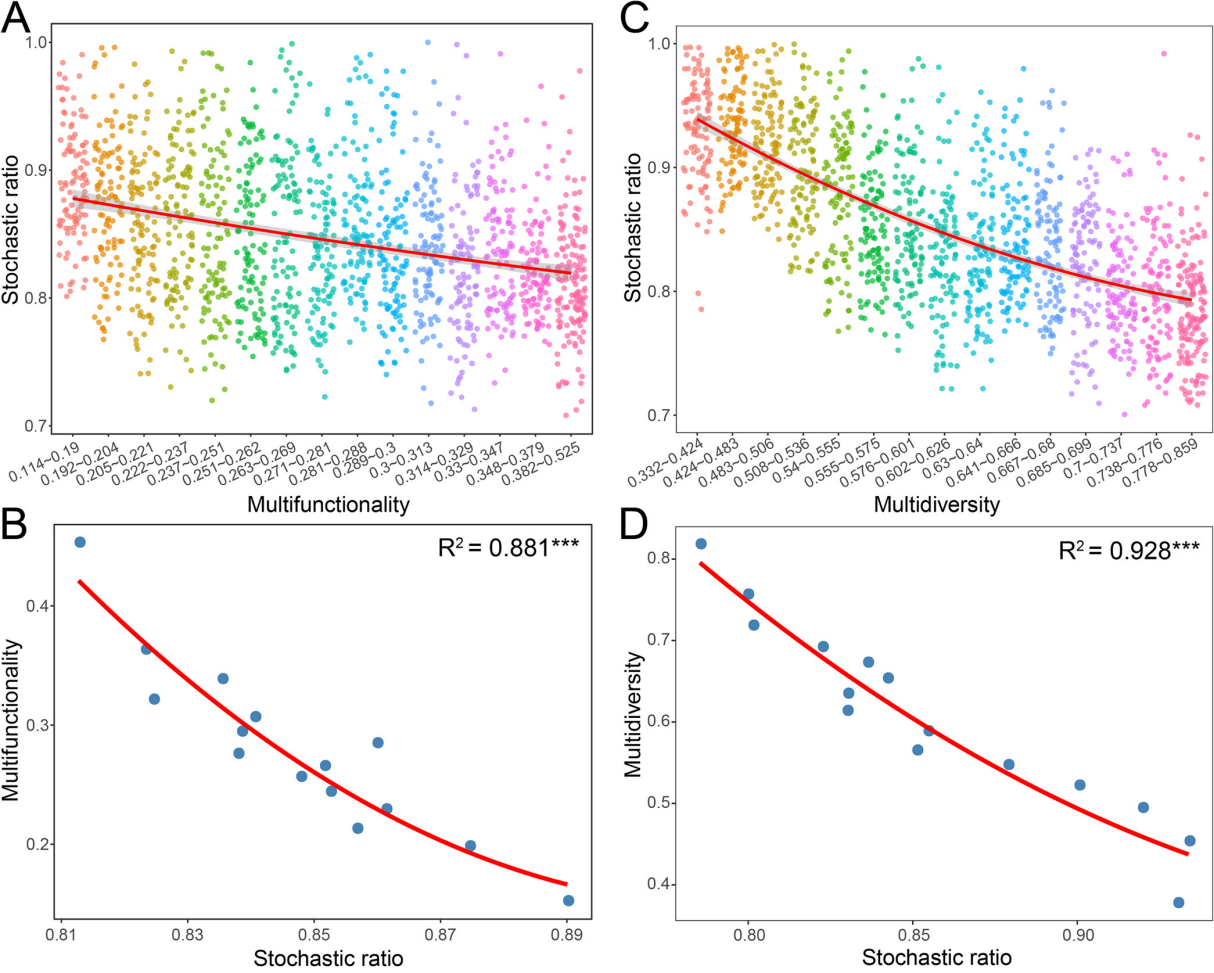
Effects of community assembly processes on multifunctionality and multidiversity of rare species. (A and C) Patterns of stochasticity ratio, estimated via null models, across different categories of multifunctionality (A) and multidiversity (C). (B and D) Relationships between stochasticity ratio versus multifunctionality (B) and multidiversity (D) were estimated by linear least-squares regression analysis with second-order polynomial fits. ***, *P* < 0.001.

**FIG 5:**
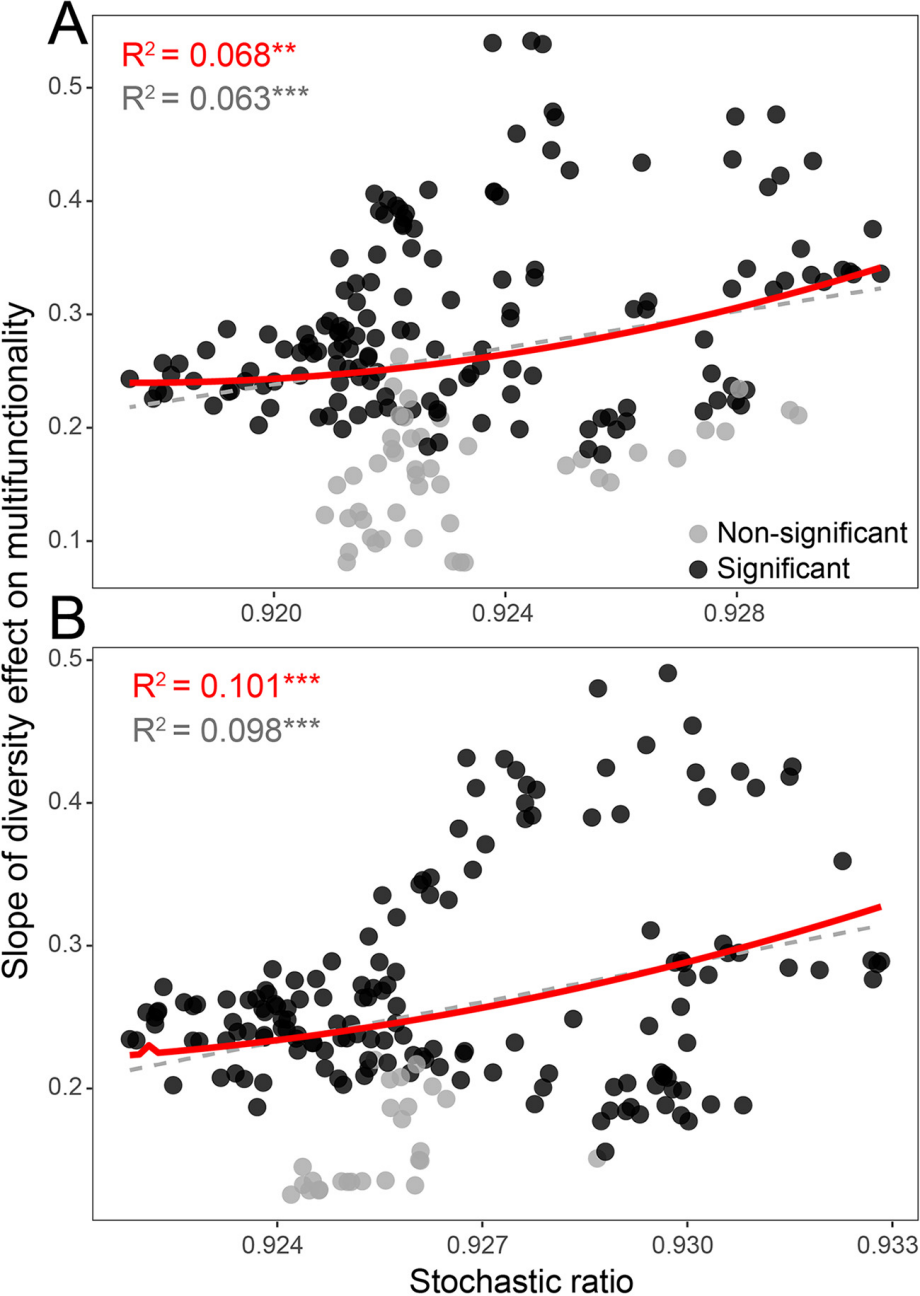
Effects of community assembly processes on the relationships between biodiversity of rare species and multifunctionality. The variances of the BEF strengths across stochasticity ratio gradients based on window sizes of 30 (A) and 40 (B) samples. Strengths of BEF relationships were estimated by the slopes of biodiversity effects on multifunctionality. Statistical analysis was performed using ordinary least-squares linear regressions with second-order polynomial fits. **, *P* < 0.01; ***, *P* < 0.001.

To explore the influence of community assembly on BEF relationships, we examined the linkages between community assembly, multifunctionality, and multidiversity. Normalized stochasticity ratio (NST) analyses revealed that the stochastic ratio of the rare subcommunity was significantly negatively correlated with multifunctionality and multidiversity (Fig. 4 and Fig. S6). However, these relationships were not observed for the abundant subcommunity (Fig. S7). This implied that community assembly processes were closely related to the ecosystem functional performance associated with the rare taxa biodiversity in soil. We further applied the moving window approach to quantify the effects of community assembly processes on the relationships between biodiversity and ecosystem multifunctionality. The results showed that the positive effects of rare taxa biodiversity on multifunctionality became stronger when the stochastic ratios increased (Fig. 5), but this was not observed for abundant taxa (Fig. S8). This result indicates that the biodiversity of rare taxa had a stronger effect on ecosystem multifunctionality when the influence of stochastic assembly processes was stronger.

10.1128/mbio.00449-22.6 FIG S6 Effect of community assembly processes on multifunctionality and multidiversity of rare species. Patterns of stochasticity ratio, estimated via null models, across the different categories of multifunctionality (A) and multidiversity (C), based on separating into 19 groups. Relationships between stochasticity ratio versus multifunctionality (B) and multidiversity (D) were estimated by linear least-squares regression analysis with second-order polynomial fits. ***, *P* < 0.001. Download FIG S6, TIF file, 1.7 MB. Copyright © 2022 Zhang et al. 2022 Zhang et al. https://creativecommons.org/licenses/by/4.0/ This content is distributed under the terms of the Creative Commons Attribution 4.0 International license.

10.1128/mbio.00449-22.7 FIG S7 Effect of community assembly processes on multifunctionality and multidiversity of abundant species. Patterns of stochasticity ratio, estimated via null models, across the different categories of multifunctionality (A) and multidiversity (C). Relationships of stochasticity ratio with multifunctionality (B) and multidiversity (D) were estimated by linear least-squares regression analysis. Download FIG S7, TIF file, 1.8 MB. Copyright © 2022 Zhang et al. 2022 Zhang et al. https://creativecommons.org/licenses/by/4.0/ This content is distributed under the terms of the Creative Commons Attribution 4.0 International license.

10.1128/mbio.00449-22.8 FIG S8 Effects of community assembly processes on the relationships between biodiversity of abundant species and multifunctionality. (A to B) Variances of the BEF strengths across stochasticity ratio gradients based on window sizes of 30 (A) and 40 (B) samples. Strengths of BEF relationships were estimated by the slopes of biodiversity effects on multifunctionality. Statistical analysis was performed using ordinary least-squares linear regressions. Download FIG S8, TIF file, 0.6 MB. Copyright © 2022 Zhang et al. 2022 Zhang et al. https://creativecommons.org/licenses/by/4.0/ This content is distributed under the terms of the Creative Commons Attribution 4.0 International license.

## DISCUSSION

Global change is causing biodiversity loss across many trophic groups (22, 48) and thereby having specific effects on multiple ecosystem functions and services (2–4, 10, 49). Given that rare species are more sensitive to environmental disturbances (22, 23), it is particularly critical to assess the functional consequences of the loss of diversity of rare taxa. Thus far, these functional consequences remain unknown. In this study, by considering multitrophic biodiversity, we provide evidence that the rare and abundant species of the belowground microbiome influence ecosystem functioning in agricultural fields in fundamentally different ways. Abundant taxa tended to maintain greater numbers of functions than rare taxa, while the rare subcommunity contributed more phylotypes which supported single ecosystem functions. Importantly, community assembly processes are closely related to the ecosystem functional performance associated with the biodiversity in the rare subcommunity. Furthermore, a higher relative contribution of stochastic assembly processes promoted the positive effect of rare taxa diversity on multifunctionality. This higher contribution of stochastic processes was also coupled with lower biodiversity and multifunctionality.

### Effects of rare and abundant soil species diversity on multifunctionality.

Local ecological communities often present a skewed abundance distribution, with relatively few dominant species coexisting alongside a high number of rare species (13, 14). These rare taxa serve as limitless reservoirs of genetic diversity and may perform a disproportionate amount of functions despite their low abundance (17, 19). However, most of our knowledge on BEF relationships is still based on whole communities, and less attention has been paid to the relative functional importance of rare and abundant species. In this study, it was found that the diversity of rare species, rather than that of abundant species, was positively related to multifunctionality in agricultural ecosystems. This suggests that rare species play an important role in maintaining multifunctionality. This result was consistent with those of previous studies which focused on macroorganisms, such as grass and fish (11, 20, 21). To further test whether the partitioning of phylotypes would affect our conclusions, we examined the main observations based on two additional definitions of rare and abundant phylotypes, including relative abundances above 1% for abundant taxa and below 0.1% for rare taxa (Fig. S9), or above 0.1% for abundant taxa and below 0.01% for rare taxa (Fig. S10). The results showed a significant and positive relationship between soil biodiversity and averaging multifunctionality for rare taxa, but not for abundant taxa. The SEM showed that the positive and direct effect of rare soil taxa diversity on multifunctionality was maintained after accounting for these other key factors. The diversity of abundant taxa had no significant effect on multifunctionality. The random forest model further confirmed these observations. Similar trends of the results among these three definitions indicated the robustness of our conclusions.

10.1128/mbio.00449-22.9 FIG S9 Effects of the biodiversity of rare (relative abundance below 0.1%) and abundant species (relative abundance above 1%) on multifunctionality. (A) Linear relationships between multifunctionality and the biodiversity of rare and abundant species. Statistical analysis was performed using ordinary least-squares linear regressions; ***, *P* < 0.001. (B) Structural equation model describing the direct relationship between the biodiversity of rare and abundant species and average ecosystem multifunctionality. We grouped edaphic properties into the same box in the model for graphical simplicity, which did not represent latent variables. Numbers adjacent to arrows indicate the size of the relationship effect. R^2^ denotes the proportion of variance explained. Red arrows represent significantly positive paths, and blue arrows represent significantly negative paths. Significance levels are as follows: *, *P* < 0.05; **, *P* < 0.01; ***, *P* < 0.001. (C) Random forest mean predictor importance of influencing factors for multifunctionality. Accuracy importance measure was computed for each tree and averaged over the whole forest (5,000 trees). Percentage increases in the MSE of variables was used to estimate the importance of these predictors, with higher MSE% values implying more important predictors. Download FIG S9, TIF file, 0.7 MB. Copyright © 2022 Zhang et al. 2022 Zhang et al. https://creativecommons.org/licenses/by/4.0/ This content is distributed under the terms of the Creative Commons Attribution 4.0 International license.

10.1128/mbio.00449-22.10 FIG S10 Effects of the biodiversity of rare (relative abundance below 0.01%) and abundant species (relative abundance above 0.1%) on multifunctionality. Download FIG S10, TIF file, 0.8 MB. Copyright © 2022 Zhang et al. 2022 Zhang et al. https://creativecommons.org/licenses/by/4.0/ This content is distributed under the terms of the Creative Commons Attribution 4.0 International license.

A high diversity of rare species might be more beneficial for multifunctionality than a high diversity of abundant species if the rare species are less likely to negatively affect ecosystem functions (11). In the present study, it was found that the functional trade-offs between organism groups, where some groups have positive effects and others have negative effects on multifunctionality, were less common among rare species than abundant species. In addition, the rare subcommunity contributed more supporting phylotypes than the abundant one, with respect to all of the single ecosystem functions. These results could explain the stronger positive effect of rare species diversity on multifunctionality.

Although the diversity of abundant soil taxa was not observed to have a significant effect on multifunctionality, individual abundant phylotypes still contributed to single ecosystem functions. The proportion of supporting phylotypes among abundant taxa was positively correlated with the number of functions the phylotypes supported. This indicated that abundant taxa tended to support multiple ecosystem functions. This conclusion is supported by the notion that abundant species occupy diverse niches, competitively utilize an array of resources, and drive variation in broad functional measures (15, 50). In contrast, negative relationships were found between the proportions of supporting rare phylotypes and the number of functions they supported. This suggested that rare taxa universally had narrower functional roles. Rare taxa are restricted by habitat specificity (13, 16) and can act as a “seed bank” for taxa that may become dominant under the appropriate conditions (51). In a previous study, it was demonstrated that positive interactions between rare taxa influence narrow functional measures (50). This could partially explain the higher functional importance of rare species, because they tend to be less redundant than abundant species with respect to the functional traits they possess. Therefore, rare species can support communities with more distinct combinations of functional traits (52).

Species interactions play important roles in stimulating ecosystem processes (5). Coexisting species, following niche partitioning based on distinct resources, promote positive interactions. These positive interactions can influence community functional performance (45, 46, 53). In this study, the abundant taxa were more strongly correlated with multifunctionality and had higher degrees in the correlation network. The opposite trend was observed for networks of rare taxa. This suggests that the abundant taxa could better support multifunctionality with greater connectivity, while the rare taxa were better at supporting multifunctionality with less connectivity. Given that highly connected taxa share similar habitats and environmental preferences (54, 55), more cooperation or interactions may be required among abundant taxa to execute higher functional performance. Meanwhile, rare taxa tended to be more independent in their support of multifunctionality. These results could be supported by the fact that abundant taxa with broader functional performance show similar functional overlaps, while rare taxa with narrower functional roles support more diverse functions (11, 52). Overall, our results suggest that unique components of complex soil communities are associated with distinct ecosystem functional performances. The findings highlight the importance of rare species for ecosystem multifunctionality, which will help guide future conservation priorities.

### Linking community assembly to BEF relationships.

Biodiversity is a core research topic in ecology because dramatic biodiversity loss could reduce ecosystem functions and services (56). Understanding the relationship between biodiversity and ecosystem functioning, and characterizing their determining factors, are central issues in ecology (29). Species assemblages are inherently important for supporting ecosystem function (57). Thus, it is critical to reveal the contributions of different assembly processes and their consequences for ecological properties and ecosystem functioning (44). It should be noted that rare taxa are major contributors to total biodiversity, meaning that the rare biosphere possesses high taxonomic diversity characterized by various functions (21); this may increase the functional redundancy of the community (i.e., the insurance hypothesis) and enable the ecosystem to respond to and counteract disturbances. The rare biosphere may act as a diverse source pool to enhance the resistance or resilience of soil microbiota and play a potential role in maintaining ecosystem function and stability under land use intensification and climate change (15, 58). Therefore, revealing the linkage between community assembly and BEF relationships for rare subcommunities is particularly important. In this study, we revealed that stochastic assembly processes promoted the positive effect of diversity on multifunctionality for rare taxa, rather than for abundant taxa. This might be partially explained by the nonsignificant BEFs for abundant taxa. Stochastic processes involve random birth, death, and dispersal events. Through stochastic assembly, species tend to co-occur when random changes and increased influxes of species are not associated with environmentally derived fitness (59, 60). In the case of this study, stochastic processes may have induced more species competition, reducing the diversity of rare taxa. The rare taxa, which contributed most of the total biodiversity, are generally vulnerable to stochastic processes, e.g., ecological drift (61). In a previous study, it was revealed that higher dispersal led to reduced biogeochemical function (62). Immigration, induced by a great contribution of stochastic processes, could bring new species from the regional pool which carry traits affecting ecosystem functioning that were not present in the initial community. In this way, stochastic processes could increase the effect of biodiversity on functions through sampling effects (43).

Deterministic processes involve nonrandom and niche-based mechanisms (63), including environmental filtering and interspecific interactions. A higher relative contribution of deterministic processes (and thus lower importance of stochastic processes) can decrease the biodiversity effect on ecosystem function via dilution. This occurs if the species selected by deterministic processes are not the species affecting ecosystem functioning (43). Furthermore, a previous study demonstrated that there were more co-occurrence associations within the soil microbiome when community assembly was primarily driven by stochastic processes (such as dispersal limitation) (64). In addition, positive species coexistence can enhance ecosystem functioning (65) and increase community functional performance due to niche partitioning by distinct resources (45, 46). Strongly hierarchical competitive community networks comprised of strong competitors have been shown to exhibit negative diversity-function relationships (29). In addition, higher relative contributions of stochastic assembly processes reduced the diversity of rare taxa and multifunctionality. This reduction may consequently reduce the effects of functional redundancy and enhance the strength of biodiversity effects. Thus, these previous studies could support our conclusion that a higher relative contribution of stochastic assembly processes promotes stronger BEF relationships. Uniquely, our study elucidates the linkage between community assembly processes and BEF relationships for rare taxa. The results extend our understanding of the mechanisms underpinning the relationships between biodiversity and ecosystem functions.

**FIG 6:**
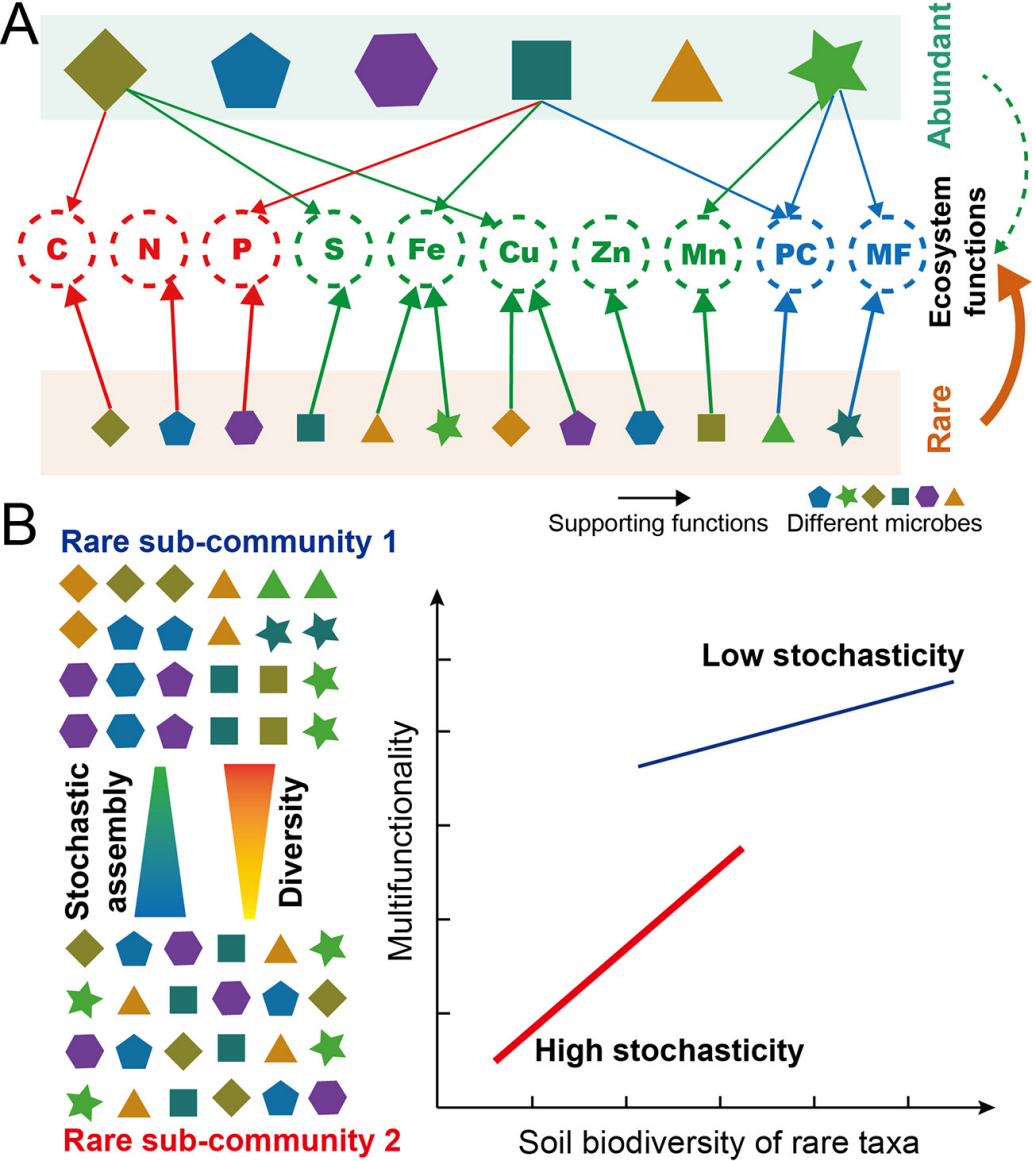
Conceptual paradigm showing the different fundamental roles of abundant and rare species in influencing ecosystem functioning (A), including functions relating to nutrient provisioning (“C,” “N,” and “P” cycles), element cycling (“S,” “Fe,” “Cu,” “Zn,” and “Mn”), pathogen control (“PC”) and plant-microbe symbiosis (“MF”), and the linkage between community assembly and BEF relationships (B). Briefly, rare taxa play major roles in maintaining ecosystem multifunctionality. Abundant taxa tended to maintain greater numbers functions than rare taxa, while the rare subcommunity contributed more phylotypes which supported single ecosystem functions. Community assembly processes are closely related to the ecosystem functional performance of soil biodiversity in the rare subcommunity. The stronger influence of stochastic assembly processes promotes the positive effect of rare taxa diversity on multifunctionality. Simultaneously, increased stochastic contributions lead to reduced rare taxa diversity and multifunctionality.

We propose a conceptual paradigm to describe the different fundamental roles of abundant and rare taxa in influencing ecosystem functioning (Fig. 6A). Briefly, rare taxa make major roles in maintaining ecosystem multifunctionality. Abundant taxa tended to maintain greater numbers of functions than rare taxa, while the rare subcommunity contributed more phylotypes which supported single ecosystem functions. An addition model shows the linkage between community assembly and BEF relationships for the rare subcommunity (Fig. 6B). Community assembly processes are closely related to ecosystem functional performance of the soil biodiversity in the rare subcommunity. A stronger influence of stochastic assembly processes promotes the positive effect of rare taxa diversity on multifunctionality. Simultaneously, increased stochastic contributions lead to reduced rare taxa diversity and multifunctionality. This study represents an exploratory effort to gain a predictive understanding of the fact that unique components of complex communities are associated with distinct functional performance. By uncovering the intrinsic linkages between assembly processes and BEF relationships, this study highlights the importance of community assembly processes in sustaining the effect of biodiversity on multiple functions of terrestrial ecosystems. These results thus help in anticipating the functional consequences of compositional changes in communities across multiple trophic groups that may be caused by land use intensification and climate change.

## MATERIALS AND METHODS

### Soil sampling and data collection.

Soil samples were collected between July and September 2017 from 114 maize and 114 rice agricultural fields. The sampled fields were adjacent to each other and less than 5 km apart. The sampling sites extended from 18.30°N to 48.35°N and 87.61°E to 99.91°E across eastern China. Thus, from south to north, the sampling sites covered tropical wet and dry climates, warm temperate climates, warm summer continental climates, and hot summer continental climates. Standard testing methods were adopted for measuring soil pH, cation exchange capacity (CEC), total organic C, and the percentage of clay, as previously described (66–68).

Soil archaeal, bacterial, fungal, and protistan communities were analyzed using high-throughput sequencing. The sequencing targets were the 16S rRNA region in archaea and bacteria, the internal transcribed spacer 1 (ITS1) region in fungi, and the 18S rRNA region in protists. The corresponding primer pairs used were Arch519F/Arch915R, 515F/907R, ITS5-1737F/ITS2-2043R, and TAReuk454FWD1/TAReukREV3, respectively (69, 70). The sequencing was performed on an Illumina HiSeq2500 platform (Illumina Inc., San Diego, CA, USA).

The acquired high quality sequences were split into operational taxonomic units using a 3% dissimilarity threshold. The OTUs with fewer than two sequences were removed. Taxonomic lineages were assigned using the RDP classifier within the SILVA database (release 128) for bacteria and archaea, the UNITE+INSD (UNITE and the International Nucleotide Sequence Databases) for fungi, and the PR2 (Protist Ribosomal Reference Database) for protists. The OTU tables were resampled to a minimum number of sequences from each sample: 36,880 for archaea, 27,712 for bacteria, 30,369 for fungi, and 5,393 for protists. This resampling was performed to ensure an even sampling depth within each belowground organism group. Protists were defined as all eukaryotic taxa excluding fungi, invertebrates (Metazoa), and vascular plants (Streptophyta), as described in previous studies (4, 71). Fungi were defined as all ITS taxa, excluding plant pathogens and mycorrhizal fungi, and were determined using FUNguild (72).

### Soil biodiversity analysis.

Considering the different species-abundance distributions among organisms, 0.5% and 0.05% were selected as the threshold values to define abundant and rare taxa, respectively (73–75). Briefly, OTUs with relative abundances above 0.5% of the total sequences were defined as “abundant” taxa; those with relative abundances below 0.05% were defined as “rare” taxa. In this study, richness (that is, the number of soil phylotypes) was used as a metric of soil biodiversity. Richness is the most extensively used and simplest biodiversity metric (4). To obtain a multidiversity index, we first standardized the richness of individual groups of soil organisms based on a common scale ranging from 0 to 1, and this was calculated according to the following formula: STD = (X − X_min_)/(X_max_ − X_min_), where STD is the standardized variable and X, X_min_, and X_max_ are the target variable, its minimum value, and its maximum value across all samples, respectively; we then calculated their average. This multidiversity index is generally accepted and has been used extensively in current literature on biodiversity-function relationships (3, 22, 76–78). Multidiversity was calculated for both the abundant and rare taxa.

### Ecosystem functioning measurements.

Sixteen ecosystem functions that are regulated by soil organisms and associated with a broad range of ecosystem services were included in this study. The 16 functions included nutrient provisioning (extracellular enzyme activities related to sugar degradation [β-glucosidase and saccharase]; chitin degradation [*N*-acetylglucosaminidase]; P mineralization [phosphatase]; soil dissolved organic C, N, and P availability; and microbial C and N stocks), element cycling (soil S, Fe, Cu, Zn, and Mn availability, involved in bio-electron transfer and energy exchange in the epigeosphere), resistance to plant pathogens (reduced relative abundance of fungal plant pathogens in soils), and plant-microbe symbiosis (abundance of soil mycorrhizal fungi).

Extracellular enzyme activities were measured using fluorometry as described previously (79). The soil dissolved organic C, microbial biomass C and N, and available N, P, S, Fe, Cu, Mn, and Zn were all measured using standard soil testing procedures (66–68). The relative abundance of potential plant pathogens and mycorrhizal fungi in soils was determined from sequencing analyses and by parsing the soil phylotypes using FUNguild (72). Only highly probable and probable guilds with an identified single trophic mode were used for the analysis. The inverse abundance (reduced relative abundance) of potential fungal plant pathogens was obtained by calculating the inverse of the variable ([total relative abundance of fungal plant pathogens] × −1). Because the values for ecosystem functions varied widely, all variables were standardized to a common scale ranging from 0 to 1 (as described above).

To derive a quantitative multifunctionality index for each sample, four independent multifunctionality approaches were used (80): multiple single functions (4), averaging multifunctionality index (76), weighted multifunctionality index (4) and principal coordinate multifunctionality index (81). To obtain an averaging multifunctionality index, the standardized scores (a common scale ranging from 0 to 1) of all individual ecosystem functions were averaged. The weighted multifunctionality index was weighted by four groups of ecosystem services (nutrient provisioning, element cycling, pathogen control, and plant-microbe symbiosis), such that functions from each ecosystem service contributed equally to multifunctionality (4). In addition, principal coordinate analysis was employed to examine the different dimensions of multifunctionality (81). Please note that we do not argue whether one approach is better or more appropriate than the others.

### Statistical analyses.

Ordinary least-squares linear regressions were conducted between soil biodiversity and multifunctionality for abundant and rare taxa. Furthermore, we performed Spearman correlation analyses between the diversity of individual kingdoms and single functions. To explore the influences of rare and abundant taxa on ecosystem functions, we estimated the Spearman correlations between individual phylotypes and single ecosystem functions. We defined phylotypes which were significantly (*P* < 0.01) and positively correlated with ecosystem functions as “supporting phylotypes.” We estimated the relationships between the proportion of supporting phylotypes and the number of functions they support via ordinary least-squares linear regressions.

A structural equation model was constructed to evaluate the direct link between the soil biodiversity of abundant and rare taxa and multifunctionality (averaging). The SEM was constructed after accounting for multiple drivers, such as climate (mean annual temperature [MAT]) and soil properties (soil pH, total C, and percentage of clay), simultaneously. Climatic data, including MAT, were obtained for all sampling sites from the Worldclim database (www.worldclim.org). In this study, mean annual precipitation was not considered because the water in the agricultural fields was partly sourced from artificial irrigation. All the variables were included as independent observable variables. The goodness-of-fit of the SEM was checked using the χ^2^ test, the root mean square error of approximation (RMSEA), and the Comparative Fit Index (CFI). The model had a good fit when the CFI value was close to 1 and the *P* values of the statistics were high (traditionally, >0.05) (82).

When the goodness-of-fit of the SEM was strong, we were able to interpret the path coefficients of the model and their associated *P* values. A path coefficient was analogous to a partial correlation coefficient and described the strength and sign of the relationship between two variables. The SEM was constructed using the “lavaan” package in R software (v3.5.1; http://www.r-project.org/) (83). In addition, random forest analysis was performed to identify the major driving factors of multifunctionality. To estimate the importance of the variables, the percentage increases in the mean squared error (MSE) of variables were analyzed; higher MSE% values implied more important variables. The analysis was conducted using the “rfPermute” package in R (84). The significance of the model was assessed with 5,000 permutations of the response variable, using the “A3” package in R (85).

Co-occurrence networks consisting of soil phylotypes from all four types of organisms were constructed for abundant and rare taxa, respectively. Robust correlations with Spearman’s correlation coefficients (ρ) of >0.6 or <−0.6 and false discovery rate-corrected *P*-values of <0.001 were used to construct the networks. We expected that these thresholds, which have been extensively used and are comparable across studies, would have both a mathematical and a biological meaning and reveal the organisms that were strongly correlated with each other. Networks were constructed using the “igraph” package in R (86) and visualized using the interactive Gephi platform (https://gephi.org). Pearson’s correlation coefficients were also calculated between each individual phylotype and multifunctionality to estimate the contribution of each phylotype to multifunctionality. In the networks, only nodes with Pearson’s correlation coefficients of >0 were displayed to highlight their support of multifunctionality.

Null model analysis was carried out using the framework described by D. Ning et al. (87) to evaluate the community assembly processes. The relative importance of stochastic processes was assessed using the stochasticity ratio (ST), which is the ratio of the mean expected similarity in the null model to the observed similarity. Because relative abundance information is useful for understanding ecological processes, dissimilarity was measured using the abundance-weighted Bray-Curtis index. The null communities were generated by randomizing the observed community structure 1,000 times based on a null model algorithm that has been previously described (88). Note that we did not chose null-model-based phylogenetic β-diversity metrics, namely, the β-nearest taxon index, to assess community assembly. The fungal ITS used in this study is a variable region and virtually impossible to align across distant fungal lineages. The phylogenetic tree would not be able accurately recover the relationships across the taxa, therefore it is difficult to use ITS for phylogenetic analysis.

The moving window approach was used to analyze the influence of community assembly on BEF relationships. This technique facilitates the analysis of multivariate data along an ecological gradient (89, 90). To ensure adequate data amounts, two window sizes of 30 and 40 consecutive samples were selected across sites, leading to the generation of 199 (1 to 30, 2 to 31… 199 to 228) and 189 (1 to 40, 2 to 41… 189 to 228) data points, respectively. The window was advanced across the sampling sites, after reordering along the latitude gradient, to generate adjacent sampling subsets to better estimate the community assembly and BEF relationships. Based on each sub-data set, we estimated the slopes of the effects of biodiversity on multifunctionality to reflect the strength of BEFs (34). Furthermore, we calculated the stochasticity ratio as described above.

### Data availability.

The raw sequence data reported in this paper are available in the NCBI Sequence Read Archive under BioProject PRJNA544819.
