# Supplementary figures and images for: Rare Species-Driven Diversity–Ecosystem Multifunctionality Relationships are Promoted by Stochastic Community Assembly
Source: mBio. 2022 Apr 14;13(3):e00449-22. doi: 10.1128/mbio.00449-22 (PMC9239226; doi:10.1128/mbio.00449-22)

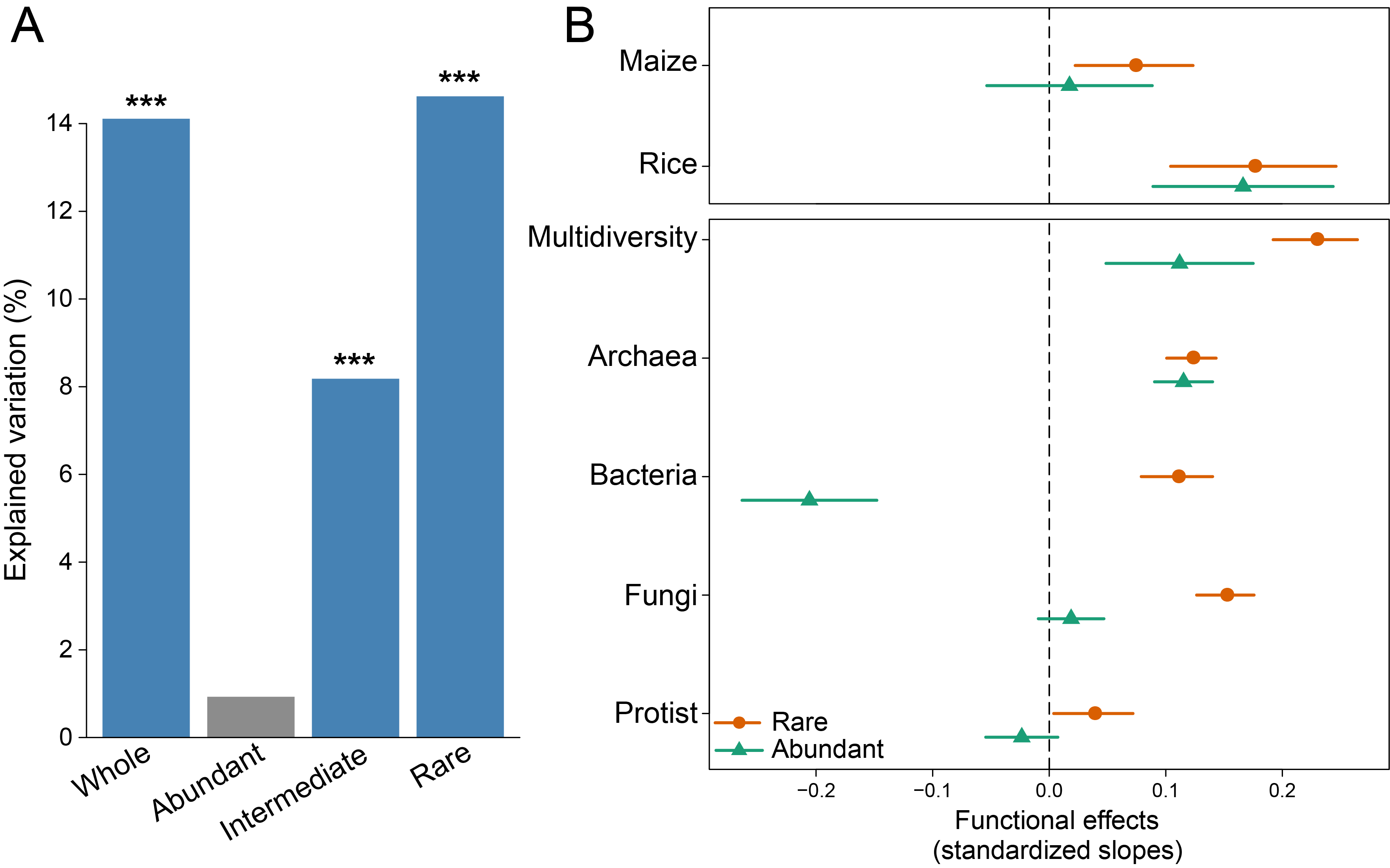

Supplement: FIG S1 [file mbio.00449-22-s0001.tif]

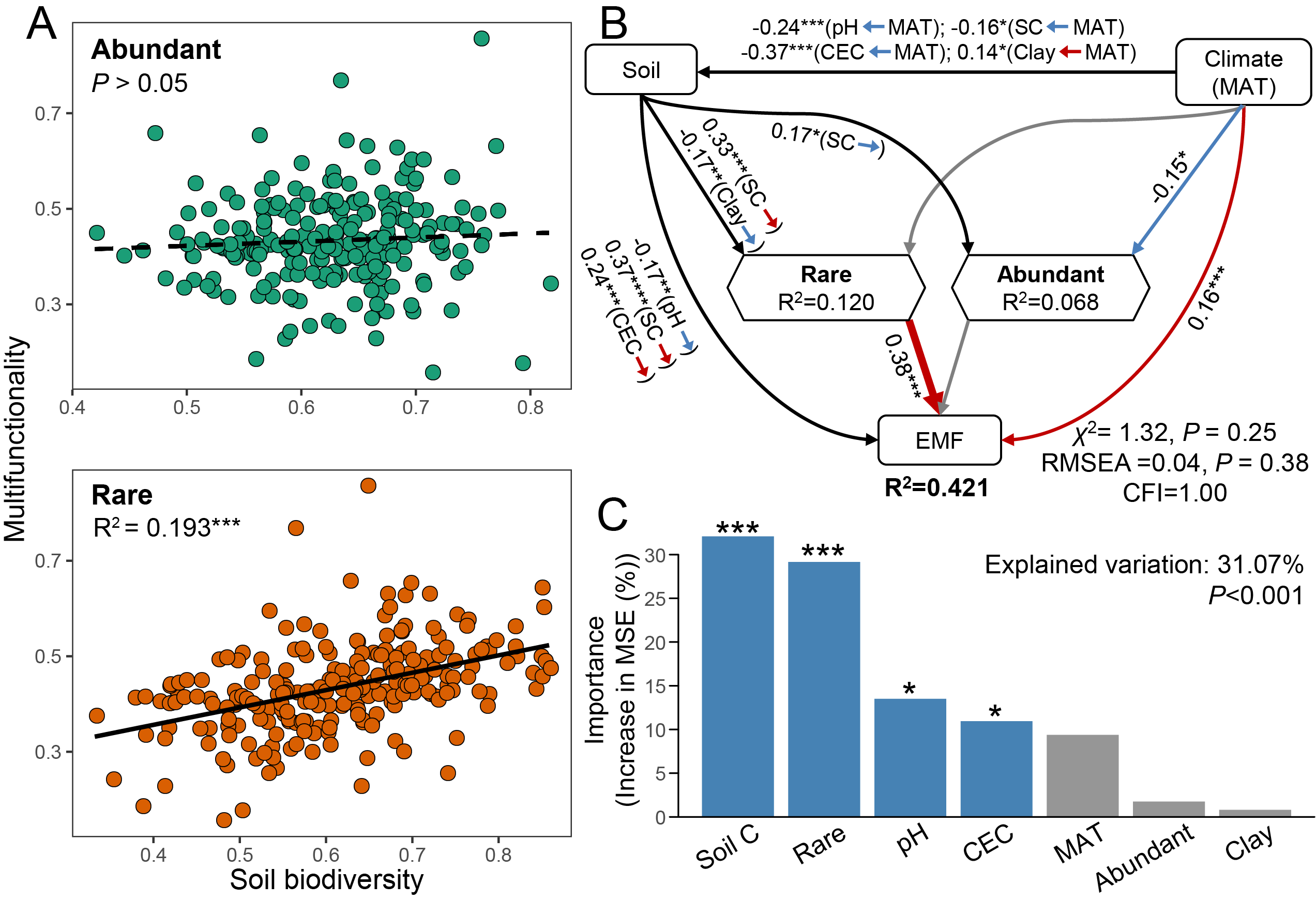

Supplement: FIG S2 [file mbio.00449-22-s0002.tif]

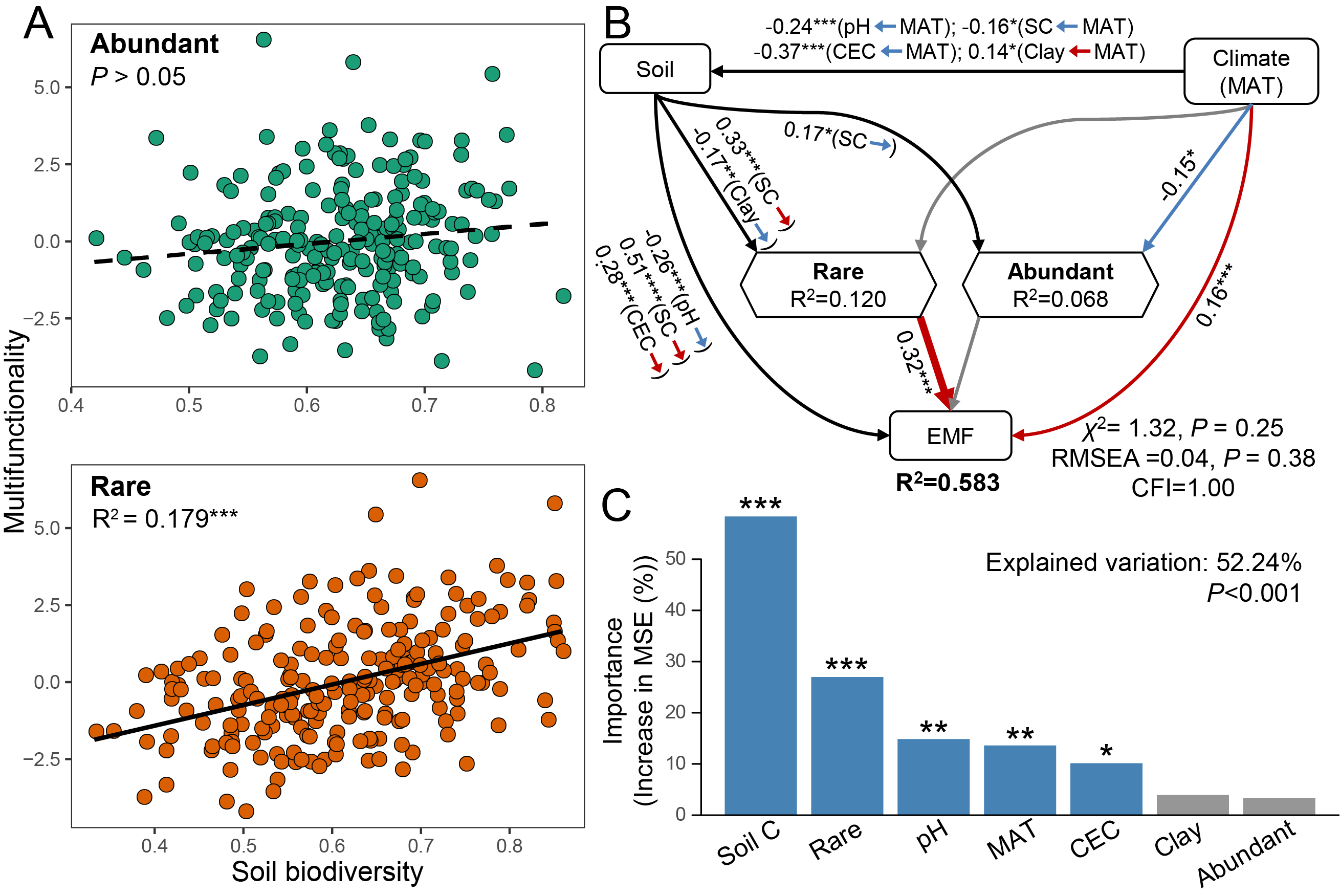

Supplement: FIG S3 [file mbio.00449-22-s0003.tif]

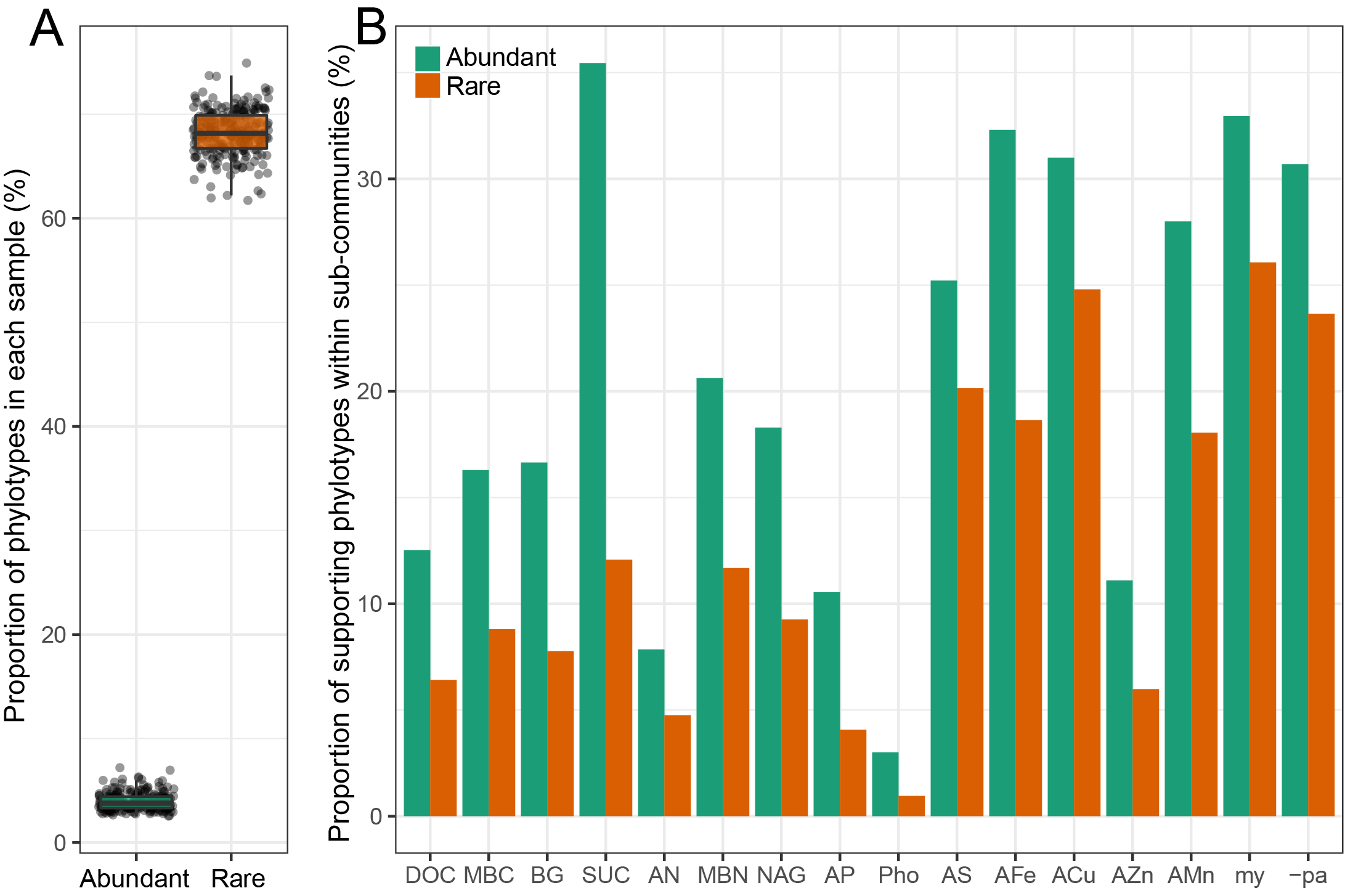

Supplement: FIG S4 [file mbio.00449-22-s0004.tif]

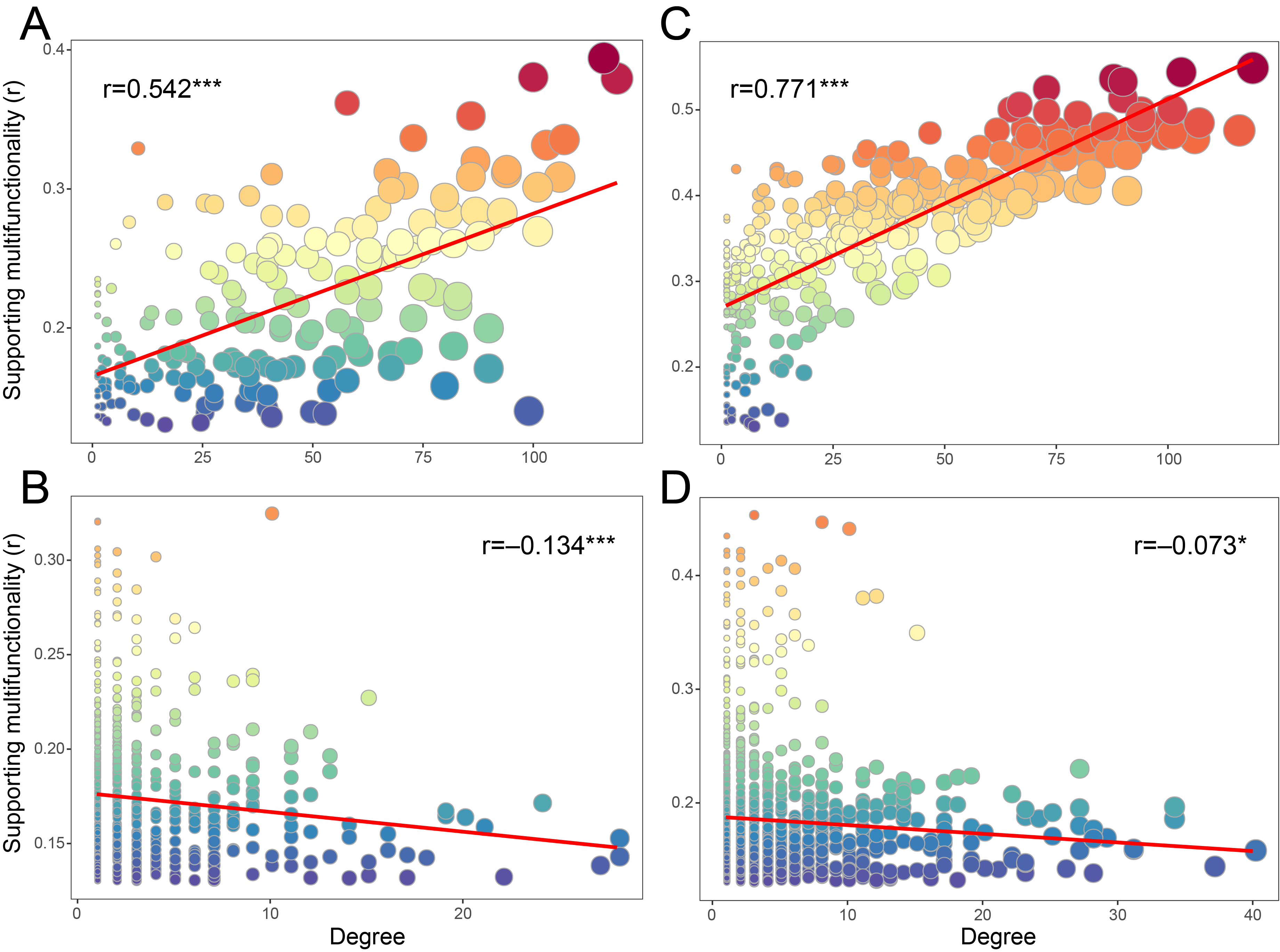

Supplement: FIG S5 [file mbio.00449-22-s0005.tif]

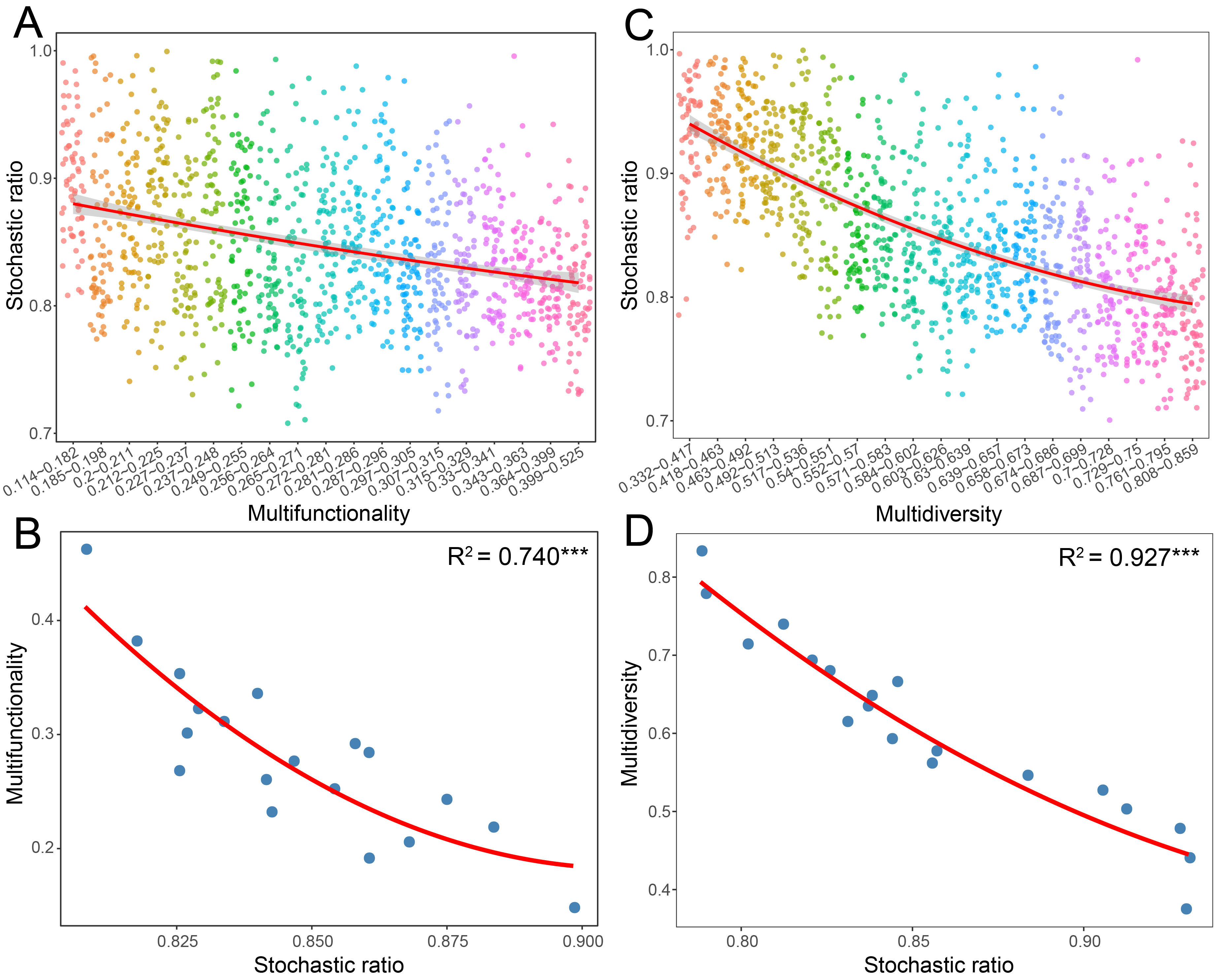

Supplement: FIG S6 [file mbio.00449-22-s0006.tif]

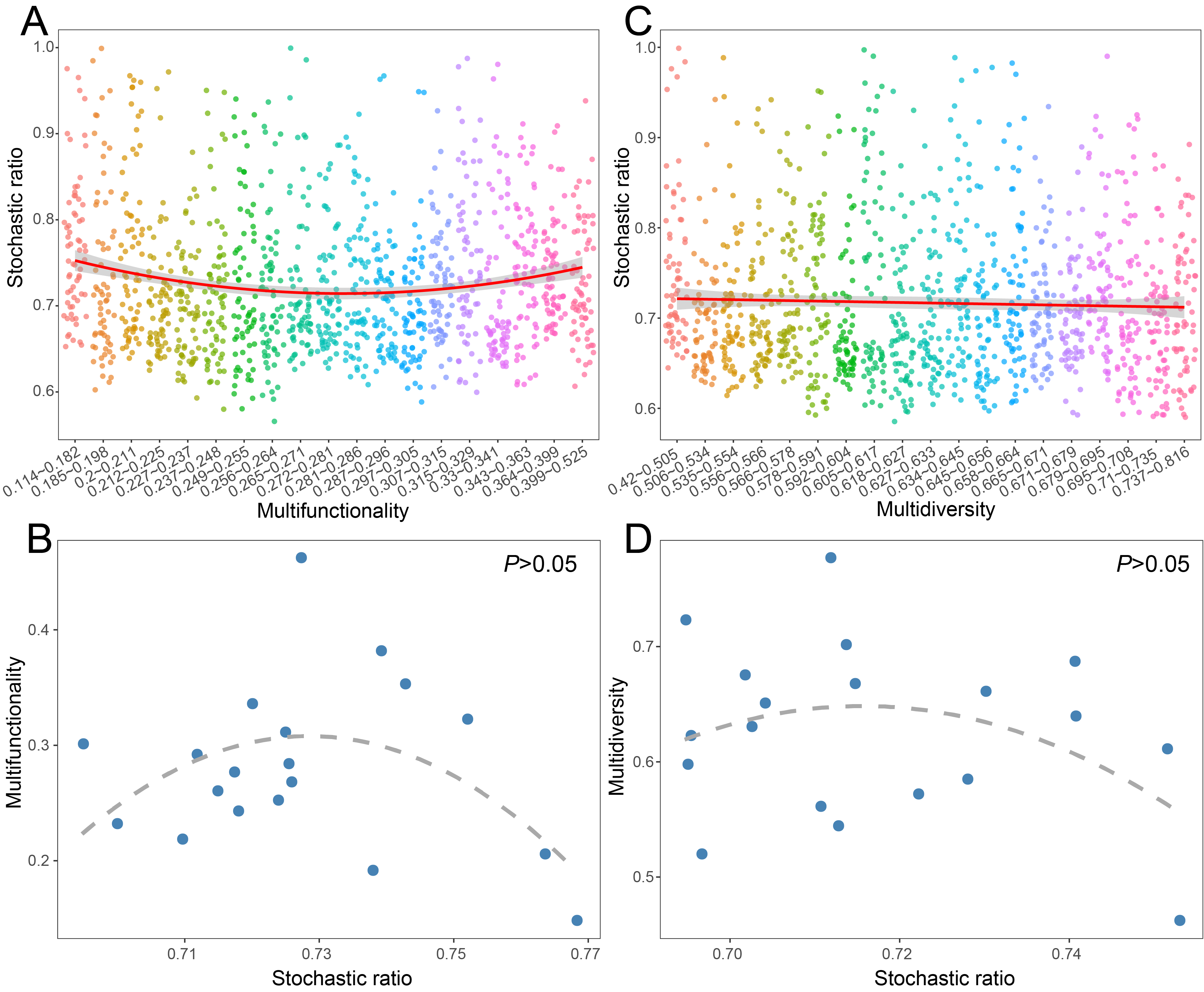

Supplement: FIG S7 [file mbio.00449-22-s0007.tif]

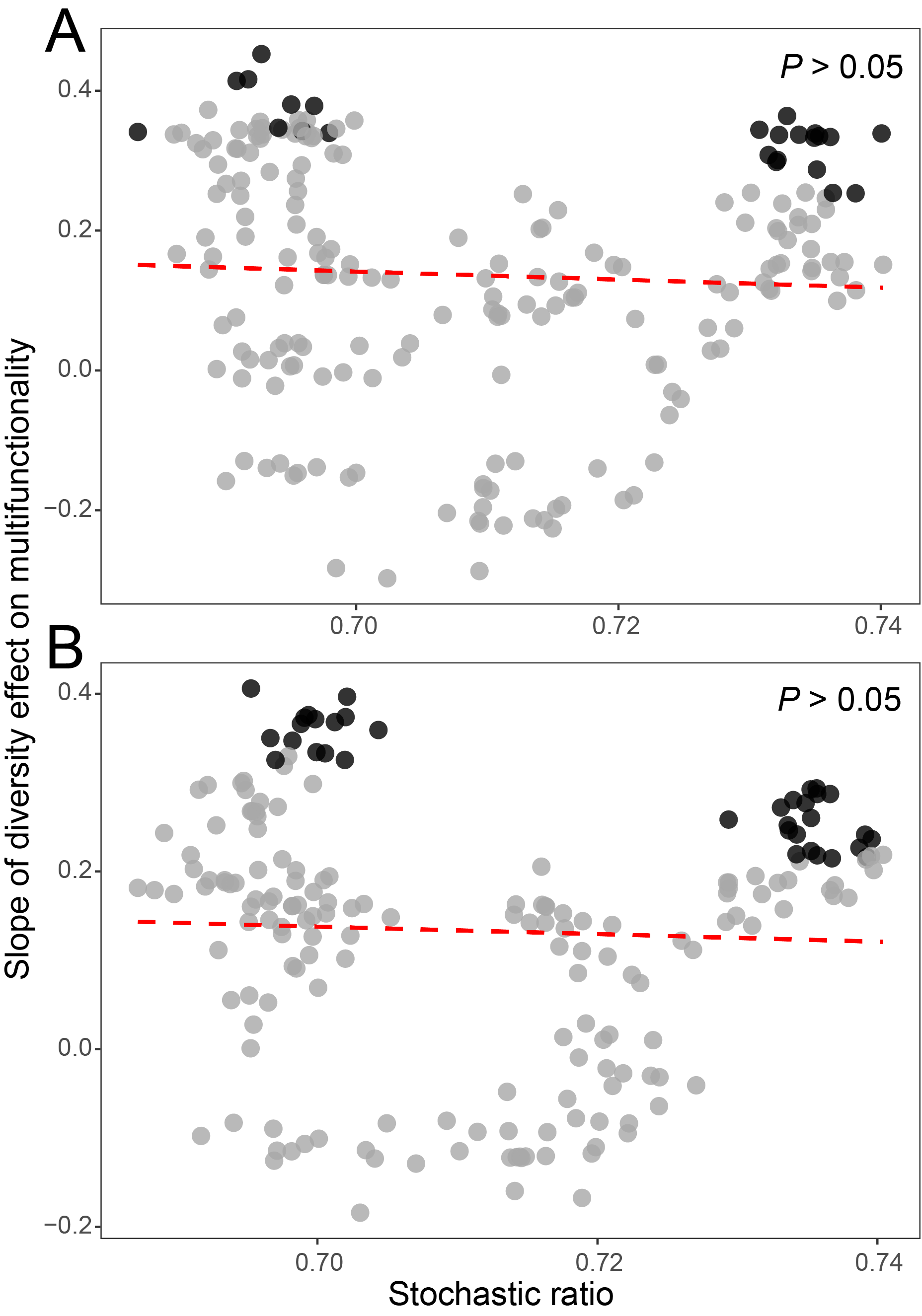

Supplement: FIG S8 [file mbio.00449-22-s0008.tif]

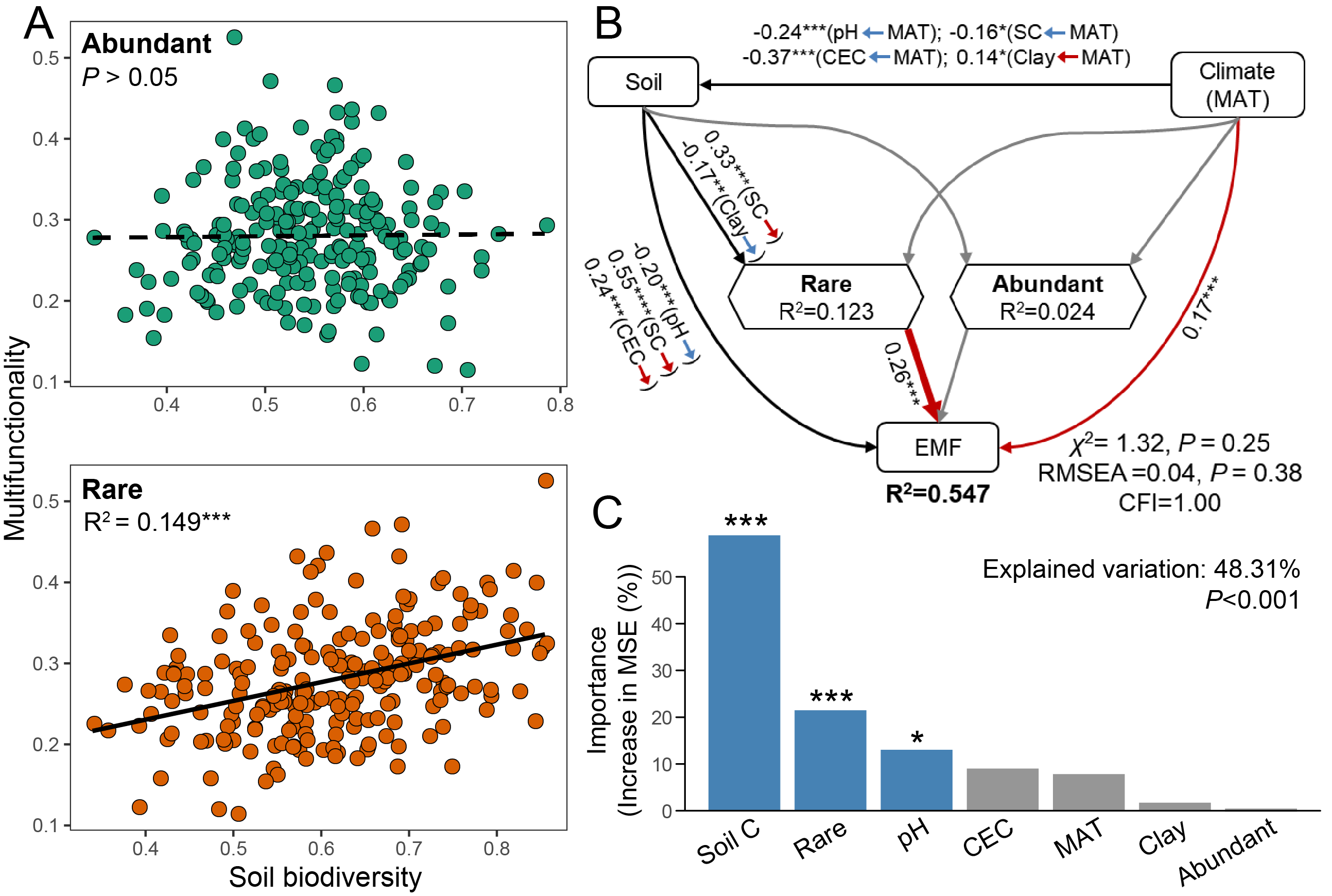

Supplement: FIG S9 [file mbio.00449-22-s0009.tif]

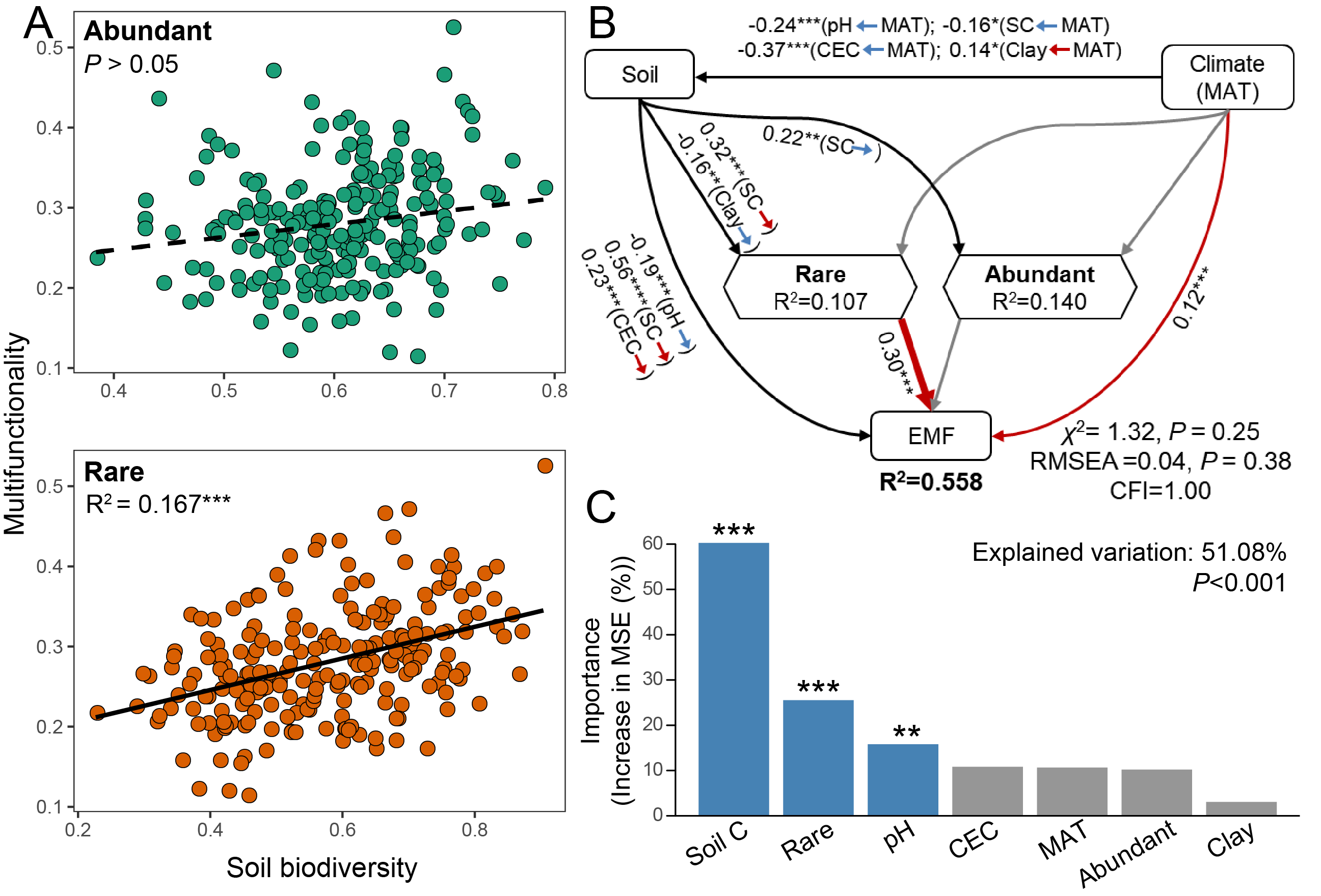

Supplement: FIG S10 [file mbio.00449-22-s0010.tif]
